# A Systematic Review on Add-On Psychotherapy in Schizophrenia Spectrum Disorders

**DOI:** 10.3390/jcm12031021

**Published:** 2023-01-28

**Authors:** Martina Nicole Modesti, Jan Francesco Arena, Natalia Palermo, Antonio Del Casale

**Affiliations:** 1 Faculty of Medicine and Psychology, Sapienza University of Rome, Via di Grottarossa 1035, 00189 Rome, Italy; 2 Department of Dynamic and Clinical Psychology and Health Studies, Faculty of Medicine and Psychology, Sapienza University of Rome, 00189 Rome, Italy

**Keywords:** schizophrenia, psychotherapy, psychosis, psychotic symptoms, social functioning

## Abstract

Schizophrenia spectrum disorders represent a varied class of mental illnesses characterised by psychosis. In addition to negative and positive symptoms, a significant lack of insight often hinders the therapeutic process. We performed an overview of the existing literature concerning these disorders to summarise the state of knowledge in the psychotherapies applied to treating psychotic symptoms. We searched the PubMed database, including randomised controlled and clinical trials, including 17 studies conducted on 1203 subjects. Psychotherapy of schizophrenia spectrum disorders can improve social functioning and positive symptoms, as well as many other symptomatic areas, and could therefore be considered a helpful adjunctive treatment of schizophrenia spectrum disorders. Among cognitive-behavioural therapies and the newest derived approaches, there is evidence that they can improve different psychotic symptoms. On the other hand, psychodynamic psychotherapies can have a positive influence on psychotic symptoms as well. Further studies are needed to identify better-tailored treatment protocols for schizophrenia spectrum disorders.

## 1. Introduction

Schizophrenia spectrum disorders represent a class of disorders burdened by severe and disabling mental illness. These are believed to have a multifactorial aetiology and embrace various clinical presentations. The main aim of treatment is to guarantee a satisfactory quality of life for patients and their families. Curing and providing care do not always coincide. On the one hand, curing, intended as a life-long remission of symptoms, may not be within reach; on the other hand, providing care can allow a great deal of improvement.

The debate over the treatment of psychosis has stemmed from so-called “biological interventions”, following a pathway that we can summarise in four steps: (1) “sleep therapy”, (2) “electroconvulsive therapy” (ECT), (3) “psychosurgery” and, finally, (4) pharmacotherapy. Psychotropic drugs are still one of the most critical fields of research in modern psychiatry, unveiled in 1952 with the discovery of the antipsychotic effects of chlorpromazine. Since the discovery of chlorpromazine’s antipsychotic effects, drugs have been crucial in our treatment protocols, but this does not mean that previous steps have been abandoned. On the contrary, they still make a field of renewed interest.

While psychiatry explored biological interventions, with the birth of psychology a new debate over the use of psychotherapy for psychosis was taking sway [1]. In this sense, research should focus on the specific role of psychotherapy in psychoses as well [2].

Sigmund Freud [3] did not believe that psychoanalytic practice would have been possible in this kind of illness, due to the inability of psychotic patients to establish transference, which is essential for therapy. Several attempts were made in this direction, but such practice gradually declined with the introduction of drugs and, moreover, it was discouraged in the 1960s by two influential studies [4,5,6] outlining a non-optimistic perspective for this kind of treatment [7].

Despite the initial discouraging premise, several of the following streams in psychology hypothesised possible aetiological pathways leading to psychotic symptoms. Progressively, starting from the 1980s, the increasing evidence for psychological, social, and environmental factors affecting psychotic symptomatology were explored and, together with genetic predisposition elements such as temperament, personality, trauma, and cognitive factors entered the scene. A new assumption joined the previous concept of psychosis as merely caused by unknown biological alterations; the way a subject makes sense of an event and the factors related to cognitive functioning are crucial in defining the subjective experience. This was the basis of the cognitive behavioural therapy (CBT) approach, and encouraged a reflection not only on therapy but also on the concept of psychosis [8]. 

Consequently, new therapeutic approaches were suggested, investigated, and nowadays appear to be framed into a vast scenery of therapeutic possibilities. Most of the evidence in psychotherapy research comes from CBT, which still accounts for the predominant number of randomised controlled trials [9].

Different studies focused on add-on psychotherapies in schizophrenia spectrum disorders, but mostly and/or exclusively on specific aspects, e.g., the effectiveness in treating positive symptoms [10], improving patient functioning [11], use of tele-psychotherapy [12], or specific interventions such as social cognition and interaction training [13].

Considering the lack of reviews focused on the global effectiveness of psychotherapies in treating symptoms of patients affected by schizophrenia spectrum disorders, the present study assessed the role of psychotherapies in addition to the standard drug treatment and aimed to summarise this field from a multifaceted perspective.

More specifically, we considered the studies that investigated the major psychotherapeutic interventions, particularly CBT and Psychodynamic Psychotherapy (PDT). In addition, we included some of the newest approaches derived from CBT and PDT, such as Metacognitive Insight and Reflection Therapy (MERIT) and Supportive Psychotherapies (SP).

Different approaches and theories have been proposed, and each one catches a particular dimension of these disorders that every single patient could present in a personal way. Our central hypothesis is that psychotherapies can improve different aspects of psychotic symptoms. Therefore, they might find a good place as a booster of the beneficial effects provided by pharmacological therapy, even in psychotic patients.

Considering the need for tailored therapeutic projects, this study aims to provide a scientific review of the most effective currently available psychotherapeutic interventions for psychosis and, if possible, to promote the outlining of integrated treatment programs conceived in a multidisciplinary key. Another aim of this study is to underline which specific interventions can improve specific symptomatologic areas of patients affected by schizophrenia spectrum disorders.

## 2. Methods

### 2.1. Search Strategy

- On December 10th, 2022, a search was conducted on PubMed^®^ with the filters title/abstract, randomised controlled trials, and clinical trials, using the terms “psychotherapy” AND “schizophrenia” in the search bar. We did not register and prepare this study based on a pre-established protocol. We did not use automation tools in our methodological processes.

Analysis and selection of articles have been conducted with the following inclusion criteria:

- Randomised controlled trials (RCT) and clinical trials on patients with schizophrenia-spectrum disorders treated with psychotherapy with or without concomitant drug treatment;
- Samples with a diagnosis of “schizophrenia spectrum and other psychotic disorders” according to the *Diagnostic and Statistical Manual of Mental Disorders* (*DSM*) (American Psychiatric Association, 1980 [14]; 1987 [15]; 1994 [16]; 2000 [17]; 2013 [18]; 2022 [19]);
- Samples with a diagnosis of “Schizophrenia, schizotypal and delusional disorders” based on the *International Classification of Diseases and Related Health Problems* 10th revision (ICD-10, 1994) [20].

We conducted a second search on the selected articles’ references, identifying four additional suitable articles.

We created a database containing eligible articles, in which all studies with the following exclusion criteria were ruled out: 1. Studies investigating the effectiveness of psychotherapy on patients with different diagnoses; 2. Studies exclusively focused on interventions other than psychotherapy.

We did not register and prepare this study based on pre-established protocols. We did not use automation tools as additional research methods.

**Figure 1:**
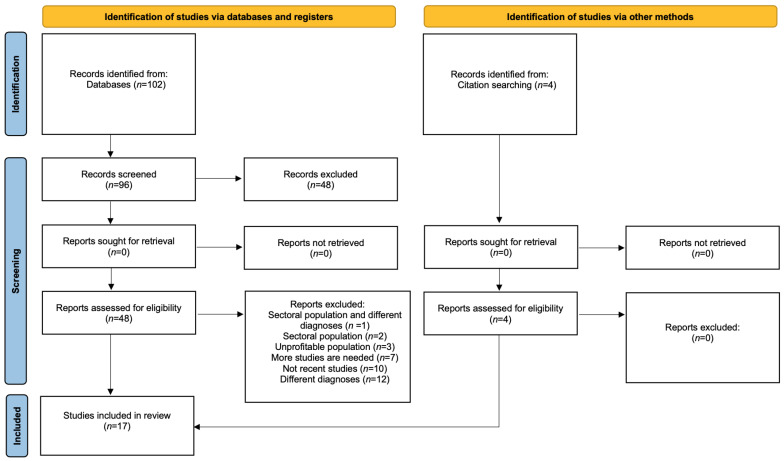
PRISMA search strategy on add-on psychotherapy of schizophrenia spectrum disorders.

We summarised the research and selection process in the PRISMA flow diagram [21], as shown in Figure 1.

### 2.2. Quality Assessment Tool and Risk of Bias

The potential methodological quality of the study and risk of bias were examined through the SIGN quality assessment tool available online [22], accessed on January 7th, 2023. All studies addressed an appropriate and focused question. Nevertheless, in all 17 studies, the two groups being analysed were selected from source populations comparable in all respects other than the factor under investigation. Even when differences were present (i.e., age, drop-out rate), they were not statistically significant. The outcomes are clearly defined in every study, the method of assessment of diagnosis is very reliable in each case (DSM), and all psychiatric diagnoses are established through these criteria worldwide. Confidence intervals have always been provided. Considering the clinical aspects, the evaluation of the methodology used, and the study’s statistical power, there is evidence of an association between the employment of psychotherapy as an adjunct treatment for psychoses and an improvement in psychotic symptomatology. In addition, according to the Grading of Recommendations Assessment, Development, and Evaluation (GRADE) system of rating in systematic reviews [23], all included studies show consistent findings with no publication bias, no severe limitations, and no severe indirectness, although they have some mild limitations that we addressed in the limitation paragraph of this review. Ultimately, considering all the included studies, their appropriateness of diagnosis as well as appropriate control of confounding, and other aspects of design, conduct, and analysis that influence the risk of bias, we can conclude that the quality of evidence is high. Ideally, future systematic reviews on this topic will comprehensively summarise evidence on the valuable role of psychotherapy in psychosis treatment.

## 3. Results

Among the seventeen selected studies, eleven sought the effectiveness of CBT as an add-on treatment of schizophrenia spectrum disorders, investigating different target symptoms and various techniques. Only one study concerned CBT within programs involving psychosocial intervention. While another single research work was based on the latter, Rakitzi et al. (2016) [24] explored the effects of integrative psychotherapy. Lastly, three studies examined different subtypes of psychodynamic psychotherapy, i.e., SP, psychodynamic group psychotherapy (PDGP), and mentalisation-based treatment for psychosis (MBT-P).

As for the origin of sources, three research works were conducted in the USA, two in the Netherlands and two in Australia. The remaining studies occurred in Croatia, Denmark, Germany, Greece, Italy, Malaysia, the United Kingdom, Scotland, Spain, and Switzerland.

The average number of participants included in every study is 70 (range 20–269), and six out of seventeen lost patients (range 0–48.58%) for several reasons, including suicide and lack of motivation.

Globally, 1203 subjects participated in the present analysis (overall drop-out rate of 5%, 59 participants). A detailed summary of all studies can be found in the following table (Table 1).

Among twelve RCTs focused on the effects of CBT, eight evaluated and proved the effectiveness in treating the positive symptoms of mindfulness-based group therapy (MGBT), cognitive behavioural social skills training (CBSST) and individualised metacognitive therapy (MCT+). Starting with the latest, conducted by Böge et al. [25], participants were randomly divided into case and control groups; the control group received the usual treatment, while the case group was subjected to four weeks of MGTB. From T0 to T1, the control group obtained significant results, although limited to positive symptoms and social skills. On the other hand, the case group improved consciousness, cognitive flexibility, and the reduction of depressive symptomatology.

De Jong et al. (2019) [26], through MERIT and Granholm et al. (2005) [27] using CBSST, demonstrated how both interventions increased metacognitive skills. Furthermore, the latter [27] confirmed a significant social functioning improvement provided by CBSST.

One study [28] investigated the effects of CBT when combined with long-term day treatment programs (DTP). Over this three-year study, 24 participants were split into cases and controls; the case group received CBT in addition to standard DTP. At the end of every year, an outcome assessment was conducted. At the end, CBT showed to improve overall symptomatology and social functioning.

Azhar et al. (2000) [29]’s research focused on reducing delusions, thanks to the combination of pharmacological therapy with CBT. Patients were divided into two groups, one was treated with risperidone, the other with chlorpromazine/haloperidol, and both groups attended CBT sessions.

Results pointed out a significant reduction in delusional beliefs and confirmed that CBT, in addition to pharmacological therapy, improves the effects of drugs. These benefits were more evident within the risperidone group, which appeared to respond faster and better compared to groups treated with other medications. This phenomenon is allegedly associated with lethargy, a common side effect that occurs less frequently in risperidone-based treatments.

The interventions carried out within Azhar’s study (*ibidem*) [29] stemmed from the assumption that “cognitive processes resulting in delusion’s formation are not different from the ones underlying non-delusional beliefs” [41,42]. The aim was to normalise delusional thoughts by reducing mental illness-related stigma to provide a change in patients’ emotional response towards their delusions.

This approach proved effective for almost every participant, regardless of pharmacological treatment. Patients themselves declared that they had found it helpful to interpret their beliefs as a result of life experiences and to consider their reactions to them understandable [29].

Moving forward, an RCT led by Sungur (2011) [36] explored the effects of psychosocial interventions associated with CBT techniques. In addition to pharmacological treatment, participants assigned to the case group were invited to attend CBT sessions and benefitted from psychosocial interventions aimed at stress reduction, anger management, and social ability improvement. Results reported a significant amelioration in social functioning for these patients.

Even supportive psychotherapy only, when coupled with usual treatment, led to a significant improvement in social functioning after one and two years from the beginning of the trial, according to Harder et al. (2014) [38].

Moreover, one study [39] outlined how PDGP benefits cognitive functions. Patients attending Group PDT sessions scored significantly higher in “spatial orientation” and “perceptual speed” than those who completed only the psychoeducational part of the program. Both clusters achieved a substantial improvement in the “ability to comprehend perceptual inadequacy”, “perceptual speed”, and capability of comprehending “relationships and connection between different situations”, even though enhancement was more evident for subjects attending group therapy sessions (ibidem) [39].

Weijers et al. (2021) [40] assumed that MBT-P, in combination with treatment-as-usual (TAU), could provide better results in social functioning than only TAU. This study included two initial sessions focused on psychoeducation, during which critical aspects of MBT-P were presented to the patients. After that, individual psychotherapy was planned to establish an excellent patient–therapist alliance, followed by closing group sessions at the end of the program. Since patients affected by psychosis experience a great deal of stress in social situations, group therapy provides the opportunity to practice mentalisation in a “stressful” environment. Both case and control groups showed significant achievements in social functioning after eighteen months, after which the program ended. Notably, only the MBT-P group maintained the achieved benefits up to a 6-month follow-up. This study did not bring up relevant differences between cases and controls regarding modifying psychotic experiences, substance abuse or positive and negative symptoms.

Rakitzi et al. (2016) [24] demonstrated the possibility of obtaining significant results on negative symptoms, working memory and cognitive functions by exploring the association of integrative psychological treatment (IPT) with treatment-as-usual (TAU). For example, IPT-group patients had higher scores concerning “insight”.

All results have been summarised in Table 2.

## 4. Discussion

First and foremost, research on CBT interventions and their subtypes has confirmed the initial assumption: psychotherapies can corroborate the effects of pharmacological treatments. Since they can influence the overall psychotic symptomatology, their use represents a valid path to improve the patient’s living conditions. Specifically, an evident effect on aspects of positive symptoms has come up, given that delusional anguish, hallucinations and their frequency turned out to be significantly diminished [31].

Seven studies proved the effectiveness of psychological interventions on positive symptoms regarding CBT and two of the “third-wave cognitive behavioural therapies” (MCT+ and MGBT): these findings meet the results of a notable network meta-analysis conducted on moderately ill patients affected by schizophrenia in 2018 [10]. The primary outcome of the study above focused exclusively on positive symptoms as the “core of the disorder”; according to our results, these were the most investigated, second only to social functioning.

On the other hand, only two studies focused exclusively on negative symptoms, similarly referred to as “the core symptom cluster of schizophrenia” in a more recent meta-analysis focused on the positive effects of social cognitive interaction training (SCIT) on social function conducted by Tang et al. (2022) [13]. The conception of schizophrenia is changing; “the core”, if still valid as a metaphor, seems to include both positive and negative symptomatic dimensions, as the latter is still essentially unapproachable with antipsychotic medications and sometimes even a consequence of some medical treatments. In this regard, the domain of negative symptoms is varied and crucial in the psychopathology of schizophrenia spectrum disorder, as it is present since the prodromal stage and persists in the inter-critical phases, forming a central area of interest for psychotherapies. In fact, according to our findings, CBT was associated with the improvement of negative symptoms and social functioning [32]. Benefits concerning anxiety, depression, social functioning and positive and negative symptoms were also observed in clinical populations undergoing MGBT.

Nevertheless, while research tends to break down the disorders focusing on specific dimensions/symptoms, a more global vision is still necessary for clinical practice. Further treatment protocols should address patients to specific interventions, considering the more disabling dimensions in each case, in addition to personal and material resources.

As for “reflective function” and the improvement of metacognition, De Jong et al. (2019) [26] found that MERIT techniques have a valid place in therapy for psychotic patients. To further highlight the merit of such interventions, patients themselves declared they had achieved a significant self-comprehension in the last-mentioned study, to such an extent that they considered the program beneficial. Similarly, MCT+ has been demonstrated to produce relevant and sustainable improvements in delusional beliefs and positive symptoms; a previous study reported consistent results by Andreou et al. [34]. These findings somewhat challenge the idea that delusions are “incomprehensible” and intrinsically resistant to rational counterarguments, opening a more wide-ranging approach to the treatment of psychoses and to patients themselves. Furthermore, a brief CBSST was validated as an intervention beneficial for “social cognition” and social functioning of psychotic people based on empirical evidence [33]. Further studies confirmed that psychosocial interventions, whether in association with CBT [36] or not [37], improved social functioning. The studies on integrative treatments showed significant improvement in negative symptoms, social functioning, and cognitive functions, especially regarding working memory [24], underlining the potential benefits that the introduction of such intervention in our treatment protocols could provide in such an impactful area.

With respect to social functioning, our results are in line with a previous systematic review and meta-analysis that investigated the effects of psychological treatments on this aspect in patients affected by schizophrenia. The lack in the literature of an overview of the impact of these interventions on patients’ functioning is highlighted and finds our total agreement [11]. The need to clarify accurately where our interventions have their best effects results in a significant amount of information sometimes referred to as particular sub-populations, which makes it difficult to assess their real-world practicability comprehensively.

Conversely, the area of PDTs suffers from chronically missing research on the effects of such interventions if compared to CBT. In any case, despite the shortage of RCTs aimed at the relevant investigation of PDT, we still have sufficient evidence in favour of its effectiveness. In 2015 the ironical title of the article “The Empirical Status of Psychodynamic Psychotherapy—An Update: Bambi’s Alive and Kicking” [43] drew attention to the fact that PDTs are found efficacious in a wide range of disorders but that still there is a significant lack of evidence, especially in the field of schizophrenia. However, Harder et al. (2014) [38] found that SP could gradually improve social functioning for up to two years. Similarly, group PDT applied to patients affected by schizophrenia successfully influenced functions such as “spatial orientation”, “perceptual speed”, and the capability of comprehending “relationships and connection between different situations” [39]. MBTp, for its part, has a proven effect on social functioning, and this result highlights how approaches involving aspects of psychodynamic psychotherapy may represent a valid path to follow.

Lastly, evidence suggests that disease duration limits the effects of psychotherapy; the prompter the intervention, the more solid the results will be [40]. Among the implications of such a concept, we would like to underline that more robust results may be achieved if the psychotherapeutic intervention is started as soon as possible after the onset; the role of timing in psychotherapies for psychosis may represent a valid topic for further research.

It is important to underline that the research on the “place in therapy” for psychotherapies is beginning to meet new needs. The more these interventions are proven effective, the more we need to consider them properly. In an interesting study, Datta et al. focused on remote cognitive remediation psychotherapy in patients with schizophrenia. They found that it is a valid and effective alternative to in-person psychotherapy in “resource-limited settings”. Between 2010 and 2019, 18% of psychotherapy research has focused on eHealth [12], suggesting that we will, more and more, have to invest in developing therapeutic strategies which are effective and affordable. In this sense, providing information suitable for evidence-based tailored treatment must be a priority to address patients to the best treatment possible, considering every single psychopathological frame.

## 5. Limitations

Our results should be taken with caution, considering some minor limitations of the studies we analysed, mainly including the sample size, which in some cases is small and does not fully allow for generalisation of results.

Some studies only included aged patients with several-year disease duration. In contrast, others focused on treatment-resistant patients or upper-class people with good education and employment.

We outlined the mismatch between case and control groups within the deficient aspects. For example, in the study conducted by Balzan et al. (2019) [30], despite randomisation, the case group was formed by patients with more severe delusional symptoms. The duration of psychological treatments differed between the included studies, and few studies had short-term follow-ups. Further minor weaknesses concern therapists: not all clinicians had the same experience with psychotic patients and the exact adherence to research protocols.

Moreover, some authors have underlined a fundamental aspect often underestimated in this field: psychological interventions’ potential risks or harm [10]. Since most of the information reported regards the presence/absence of effectiveness in a certain dimension, it is difficult to assess whether some of these interventions may be dangerous or contraindicated for patients, especially if carried out by non-experts.

Despite these limitations, among the studies included many were characterised by excellent adherence to the initial protocol and presented high-quality standards that complied with CONSORT guidelines. In particular, the research of Böge et al. (2021) [25] met high-methodological criteria regarding blind and randomised allocation. In all the included articles, rigorous control over treatment administration and efficient daily assistance was performed so that patients declared themselves satisfied, and a low drop-out rate characterised most studies. To our knowledge, this is the first systematic review which has focused on all psychotherapeutic approaches currently employed in add-on in the treatment of schizophrenia spectrum disorders, and not only on one specific intervention and/or one specific symptom. Our results are, indeed, in line with the latest meta-analysis on this topic, overcoming its central bias of concerning only RCTs performed in China and, therefore, confirming that the results can be applied overall despite geographical and ethnological differences [44]

Further studies should manage to gather more heterogeneous and numerous samples. Moreover, the case and the control groups should differ only in the variables under evaluation, and treatment, to reach more substantial evidence for statistical analysis.

## 6. Conclusions

Psychotherapy of schizophrenia spectrum disorders can improve social functioning and positive symptoms, as well as many other symptom areas, such as described thoroughly in Table 2, and should, therefore, be regularly considered a helpful adjunctive treatment of schizophrenia spectrum disorders. Among CBTs and the newest derived approaches, there is evidence that MGBT, MERIT, social skills training (SST), and individualised MCT+ can improve different psychotic symptoms.

On the other hand, psychodynamic psychotherapies such as SP, PDGP, MBT-P, and IPT can have a positive influence on psychotic symptoms as well.

Regardless, therapeutic intervention should be designed considering every personal need and characteristic, following a patient-centred approach. Standard protocols pose the risk of neglecting individuality as a critical resource for all appropriate treatments.

Of course, this review aims to underline the importance of psychotherapy in add-on to pharmacological treatments in schizophrenia spectrum disorders, which are known not to be able to act on social functioning and/or negative symptoms specifically.

Future research should also focus on constructing empirical bases to understand the processes underlying social functioning, typically deficient in psychotic patients. Studies are now focusing on establishing tailored pharmacological therapies for schizophrenia, and the same should be carried out with respect to psychological treatments in add on. This would help to determine which interventions promote the best recovery, aiming more and more at the conception of integrative approaches. An inspiration for future research would be to integrate CBT and psychodynamic psychotherapy to investigate a tailored psychotherapeutic protocol for patients with schizophrenia.

## Figures and Tables

**Table 1:** Summary of included studies.

Intervention	Technique	Study	Cases	Controls	Pharmacological Therapy
CBT	MGBT	Böge et al., 2021 [25]	21	19	Antipsychotics: olanzapine Antidepressants: fluoxetine Mood Stabilisers: non-specified Benzodiazepines: lorazepam
CBT	MERIT	de Jong et al., 2019 [26]	Initial sample: 35 Final sample: 18		Non-specified pharmacological therapy
CBT	CBSST	Granholm et al., 2005 [27]	37	39	Antipsychotics: typical or atypical Antidepressants: *n.s.* Mood stabilisers: *n.s.*
CBT	Integrating Cognitive Behavioural Psychotherapy + DTP	Bradshaw, 2000 [28]	Initial sample: 24 Final sample: 15 Group division not specified.		Antipsychotics: phenothiazine (Thorazine) e haloperidol (Haldol).
CBT	CBT	Azhar et al., 2000 [29]	Risperidone + CBT: 10	chlorpromazine/haloperidol + CBT: 10	Antipsychotics: risperidone vs. chlorpromazine/haloperidol
CBT	MCT+	Balzan et al., 2019 [30]	MCT+: Initial sample: 27Final sample: 26	CR: Final sample: 27 Initial sample: 26	Antipsychotics: n.s.
CBT	CBT	Kuipers et al., 1998 [31]	Initial sample: 28 Final sample: 23	Initial sample: 32 Final sample: 24	Antipsychotics: chlorpromazine equivalents
CBT	CBT	Startup et al., 2004 [32]	43	47	Atypical antipsychotics: olanzapine, quetiapine, risperidone, amisulpride or clozapine
CBT	SST	Rus-Calafell et al., 2013 [33]	18	13	Antipsychotics: typical, atypical or in combination
CBT	MCT+	Andreou et al., 2017 [34]	46	46	Antipsychotics: chlorpromazine
CBT	CBT	Durham et al., 2003 [35]	CBT: 22 SPT: 23	TAU: 21	Typical antipsychotics: chlorpromazine equivalents Atypical antipsychotics: n.s.
Psychosocial intervention + CBT	OCM	Sungur et al., 2011 [36]	50	50	Antipsychotics: Olanzapine, haloperidol, or other first-generation antipsychotics
Psychosocial intervention	SST/Supportive Group Psychotherapy	Marder et al., 1996 [37]	SST: 43	SP: 37	Antipsychotics: fluphenazine
IPT	IPT	Rakitzi et al., 2016 [24]	IPT: Initial sample: 24 Final sample: 18	TAU: Initial sample: 24 Final sample: 15	Atypical antipsychotics: chlorpromazine equivalents
PDT	SP	Harder et al., 2014 [38]	119	150	Non-specified pharmacological therapy
PDT	PDGP	Restek-Petrović et al., 2014 [39]	PDGP: 18	10	Atypical antipsychotics: *n.s.*
PDT	MBT-P	Weijers et al., 2021 [40]	Initial sample: 90 Final sample: 84 MBT-P: 42; TAU: 42		Non-specified pharmacological therapy

CBT: Cognitive Behavioural Therapy; CBSST: Cognitive Behavioural Social Skills Training MGBT: Mindfulness-based Group Therapy; MERIT: Metacognitive Reflection and Insight Therapy; DTP: Day Treatment Program; MCT+: Individualised Metacognitive Therapy; SST: Social Skills Training; SP: Supportive Psychotherapy; OCM: Optimal Case Management; IPT: Integrated Psychological Treatment; PDT: Psychodynamic Psychotherapy; PDGP: Psychodynamic Group Psychotherapy; MBT-P: Mentalisation-based Treatment for Psychotic Disorder; CR: Cognitive Remediation Therapy; TAU: Treatment as Usual; NS: non-specified.

**Table 2:** Summary of results.

Research	Intervention	Technique	Effectiveness on Positive Symptoms	Effectiveness on Negative Symptoms	Depression and Anxiety	Social Functioning	Cognitive Functions	Metacognitive Abilities
Böge et al., 2021 [25]	CBT	MGBT	wing	✓	✓	✓		
de Jong et al., 2019 [26]	CBT	MERIT						✓
Granholm et al., 2005 [27]	CBT	CBSST				✓		✓
Bradshaw, 2000 [28]	CBT	Integrating Cognitive-Behavioural Psychotherapy + DTP				✓		
Azhar et al., 2000 [29]	CBT	CBT	✓ Delusions		✓			
Balzan et al., 2019 [30]	CBT	MCT+	✓					
Kuipers et al., 1998 [31]	CBT	CBT	✓					
Startup et al., 2004 [32]	CBT	CBT	✓	✓		✓		
Rus-Calafell et al., 2013 [33]	CBT	SST				✓		
Andreou et al., 2017 [34]	CBT	MCT+	✓					
Durham et al., 2003 [35]	CBT	CBT	✓ Delusions					
Sungur et al., 2011 [36]	Psychosocial intervention + CBT	OCM				✓		
Marder et al., 1996 [37]	Psychosocial intervention	SST/Supportive Group Psychotherapy				✓		
Rakitzi et al., 2016 [24]	IPT	IPT		✓		✓	✓ Working Memory	
Harder et al., 2014 [38]	PDT	SP				✓		
Restek-Petrović et al., 2014 [39]	PDT	PDGP					✓	
Weijers et al., 2021 [40]	PDT	MBT-P				✓		

## Data Availability

Not applicable.
